# The silent microbial shift: climate change amplifies pathogen evolution, microbiome dysbiosis, and antimicrobial resistance

**DOI:** 10.1186/s40794-025-00275-y

**Published:** 2025-11-13

**Authors:** Nourhan G. Naga, Radwa M. Taha, Eman A. Hamed, Enas A. Nawar, Hadeer O. Jaheen, A’laa A. Mobarak, Yasmen M. Radwan, Aya G. Faramawy, Mervat A. Arayes

**Affiliations:** 1 Department of Botany and Microbiology, Faculty of Science, Alexandria University, Alexandria, Egypt; 2 Department of Botany and Microbiology, Faculty of Science, Damanhour University, Damanhour, Egypt

**Keywords:** Climate change, Antimicrobial resistance, Microbiome dysbiosis, Pathogen evolution, Artificial intelligence

## Abstract

Climate change is a primary driver of new infectious diseases. It affects pathogen evolution, vector ecology, and human susceptibility. Rising temperatures, changed rainfall patterns, and extreme weather events contributed to the spread of vector-borne, food-borne, and water-borne diseases. Furthermore, climate stressors promote antimicrobial resistance (AMR) and disrupt the human microbiome. This increases susceptibility to infections and chronic diseases. This review explores the complex relationships between climate change, microbial ecosystems, and public health focusing on how microbial dysbiosis and environmental changes interact to influence disease dynamics. We also highlight long-term solutions, such as One Health approaches, probiotics, and AI-powered early warning systems, as strategies for reducing future risks. Addressing these challenges requires global collaboration, climate-resilient health systems, and proactive actions to mitigate the health consequences of a rapidly changing environment.

## Introduction

Trillions of different microbes live in the human body. Together they form the microbiome [1]. The living microorganisms present in a specific environment, such as the gut or oral cavity, are referred to as the microbiota. The term microbiome encompasses the collective genomes of these microorganisms. It also includes their structural components, metabolites, and interactions with environmental factors [2]. A deviation from the optimal microbial composition in habitat is known as microbial dysbiosis or microbial shift [3], can result in impaired immunity and increased susceptibility to infection. Numerous infectious disorders have been linked to microbiome dysbiosis. Some conditions arise from proliferation of pathogenic species and/or a reduction in beneficial symbionts [4].

The concept of “microbial shift” and its implications for host health, particularly in the context of infectious diseases, represents a significant paradigm shift in how we think about and manage disease. The term “silent microbial shift” refers to the often gradual and imperceptible changes in microbiome composition and function. Recent studies have shown that a host’s susceptibility to infections and the course of disease are significantly influenced by their microbiome [5, 6]. Growth, digestion, the development of the immune system proper functioning, and neural development all rely on this balance [7]. When this equilibrium is disrupted, the host becomes more vulnerable to pathogenic invasion and subsequent disease [8]. While microbial equilibrium is vital for host health, climate stressors disrupt this balance by altering environmental conditions. Microorganisms are particularly affected by climate change due to their high sensitivity to environmental fluctuations [9]. This review frames climate change as a systemic risk to microbial balance, which underlies food safety, environmental resilience, and human health. Loss of microbial balance serves as the central thread linking diverse outcomes such as antimicrobial resistance (AMR), ecosystem collapse, and human microbiome disruption.

## The underestimated link between climate change and infectious disease dynamics

The changing climate represents a serious risk to the health of humans, animals, plants, and the planet as a whole [10]. This pressing issue significantly affects all ecosystems. Global climate shifts can have profound impacts on disease dynamics within local communities, influence the likelihood of disease emergence, and facilitate the global spread of infectious diseases [11].

Ecosystems are susceptible to degradation and perhaps extinction as a result of the interplay between local human activity and climate change [12]. Climate change exerts both direct and indirect effects on human health, influenced by social, environmental, and public health determinants. The effects of climate change, especially with regard to infectious diseases, are becoming increasingly evident as its pace accelerates [13]. Rising temperatures, altered precipitation patterns, and severe weather conditions like floods, wildfires, heatwaves, and storms are associated with heightened health risks [14]. These changes result in increased mortality, a surge in non-communicable diseases, and medical emergencies, while also amplifying the transmission and prevalence of infectious diseases, posing severe threats to human health.

There are several ways that climate change affects the transmission of infectious diseases, including altered pathogen growth, vector distribution shifts, and modifications in human behavior, which collectively contribute to an increase in certain infectious diseases [11, 15]. Additionally, the migration of humans and animals, along with rising population densities, accelerates pathogen transmission [16]. Climate change also threatens biodiversity by rendering parts of current species’ ranges climatically unsuitable, forcing species to adapt, migrate, or face the risk of extinction [17].

All microorganisms and their vectors are affected by climate change, leading to an increase in pathogenic microbes that contaminate food and water. This, in turn, poses significant risks to both human and animal health by exerting direct and indirect impacts on food security [18].

According to the World Health Organization (WHO), air pollution is a major contributor to climate change. Nearly 99% of the global population is exposed to air pollutants at harmful levels [7]. Air pollution is a critical environmental health concern, as diseases associated with it are responsible for approximately 7.2 million premature deaths worldwide [19].

## Environmental changes driving pathogen emergence

### Temperature rise and pathogen survival

One of the most extensively researched and debated issues affecting the Earth’s ecosystem is global warming and climate change [20]. Climate change is predicted to increase the frequency, intensity, and duration of heatwaves and extreme temperature events [21]. Global warming accelerates the melting of glaciers and ice sheets and causes ocean expansion, resulting in rising sea levels, altered rainfall patterns, and increased flooding. Droughts and flooding create stagnant water and shorten extrinsic incubation periods, thereby contributing to a rise in vector-borne diseases [22].

Threats to human security, livelihoods, food security, water availability, health, and economic growth are expected to rise sharply with 1.5 °C of warming and even more at 2 °C. The primary driver of this temperature rise is the increase in greenhouse gas emissions, particularly carbon dioxide (CO₂) [23]. According to the Intergovernmental Panel on Climate Change (IPCC), human health will suffer from even a small increase in global warming [24].

Climate change leads to rising temperatures and altered precipitation patterns, creating favorable conditions for the growth and survival of food-borne parasites. Food-borne infections are responsible for approximately 91 million illnesses and 137,000 deaths annually in Africa [25]. Warmer temperatures can increase parasite metabolic rates, enabling faster reproduction and larger population sizes, while also intensifying bacterial, viral, and pathogenic contamination of water and food [26, 27]. All terrestrial food chains are fundamentally dependent on plants. Therefore, understanding and predicting how plant–pathogen interactions may shift as a result of climate change is crucial. To date, most plant–pathogen thermal tolerance assessments have focused on agricultural diseases [28], with growing evidence suggesting that global warming could accelerate the spread of these diseases [29].

## Melting permafrost and ancient pathogen release

Permafrost and permanently frozen regions, such as glaciers, are thought to be natural repositories of a large number of primarily inactive microorganisms, including potential human pathogens [30]. Average temperatures in the Arctic are rising at more than twice the rate observed in temperate regions, making the effects of climate change particularly evident in these areas [31, 32].

**Fig. 1:**
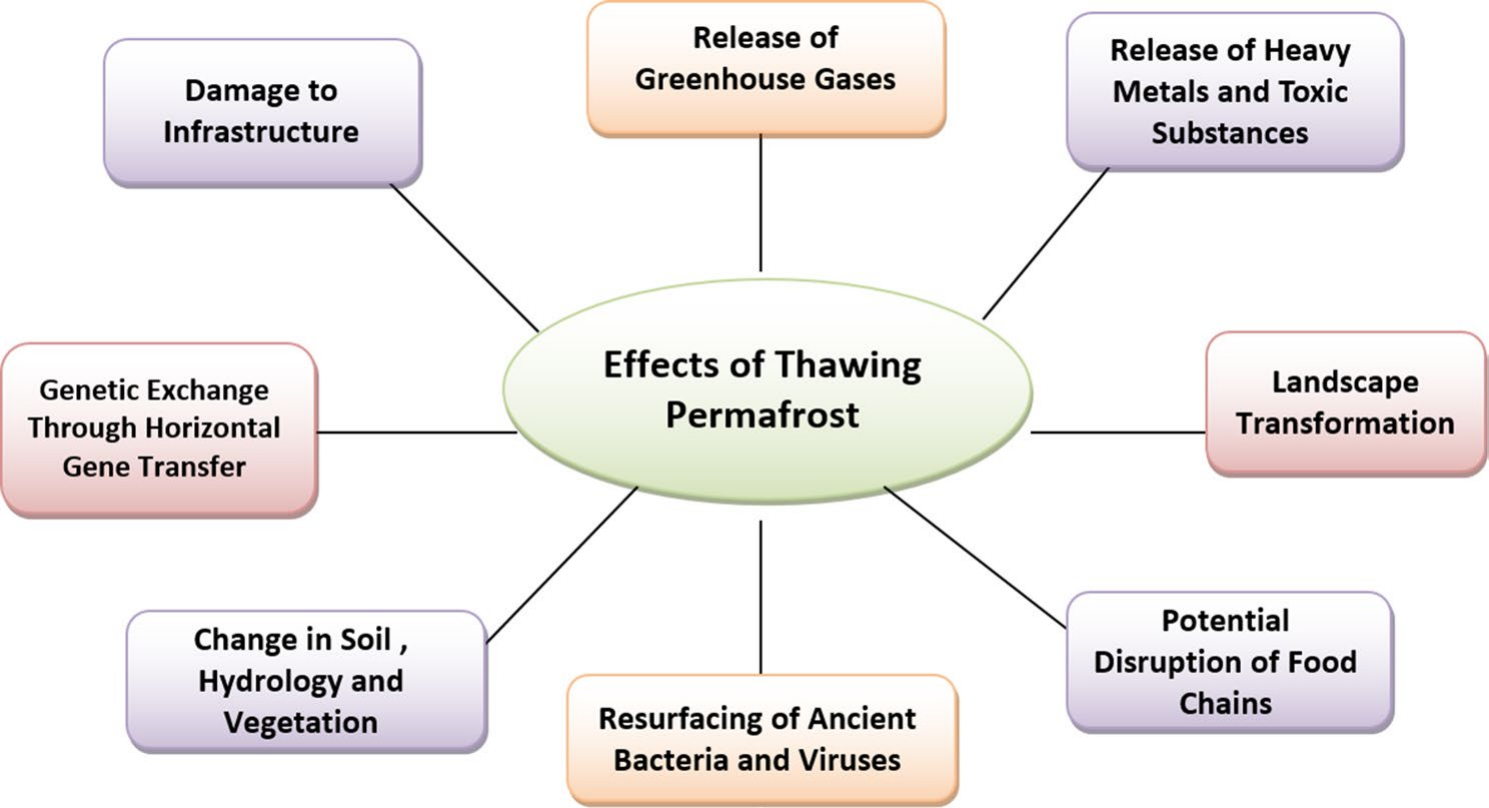
The worst effects of thawing permafrost

Global permafrost thawing at deeper layers represents one of the most serious microbiological consequences of climate change. This thawing is expected to have multiple severe implications (Fig. 1). Ancient organic material, preserved for millennia in the deep strata of permafrost, is now being released due to its rapid thawing [32]. Since 1980, climate change has steadily increased permafrost temperatures, decreasing the depth of frozen ground and thawing the outermost layers, which significantly affects its stability [33]. Once temperatures rise above freezing, the return of liquid water triggers the metabolic reactivation of various soil microorganisms, bacteria, archaea, protists, and fungi [34, 35]. There are a number of detrimental effects of this process. For example, permafrost soils in the Arctic retain over twice as much carbon as the atmosphere, and their uncontrolled release exacerbates global warming [30].

Scientists have identified pathogens in permafrost since the late 19^th^ century, including viruses and bacteria, capable of surviving in freezing conditions for thousands of years [36]. The climate crisis has heightened concerns about the potential re-emergence of ancient pathogens; indeed, a disease originating from permafrost has recently been reported to infect both animals and humans [37].

## Habitat fragmentation and human-wildlife microbe interactions

Despite decades of conservation efforts and a notable increase in protected land, habitat loss, fragmentation, and climate change continue to drive the decline and extinction of numerous species at local, regional, and global scales [38]. Fragmentation alters both biotic and abiotic conditions, reducing habitat availability and often degrading the quality of remaining habitat patches [39].

Furthermore, climate change may compel species currently residing in protected areas to migrate beyond these zones, thereby undermining previous conservation efforts [17]. The ability of species to shift their ranges in response to changing climate conditions becomes increasingly constrained when suitable habitats are scarce and fragmented within increasingly hostile landscapes [38].

Microbial interactions are significantly influenced by habitat fragmentation [40, 41]. Since microbiomes play a critical role in ecosystem functioning [42], understanding how fragmentation affects these unseen actors and their interactions is essential [39]. While the consequences of fragmentation for plants and animals have been extensively studied, its implications for microbial communities have only recently begun to be explored [43].

## Urbanization and its interplay with climate

**Fig. 2:**
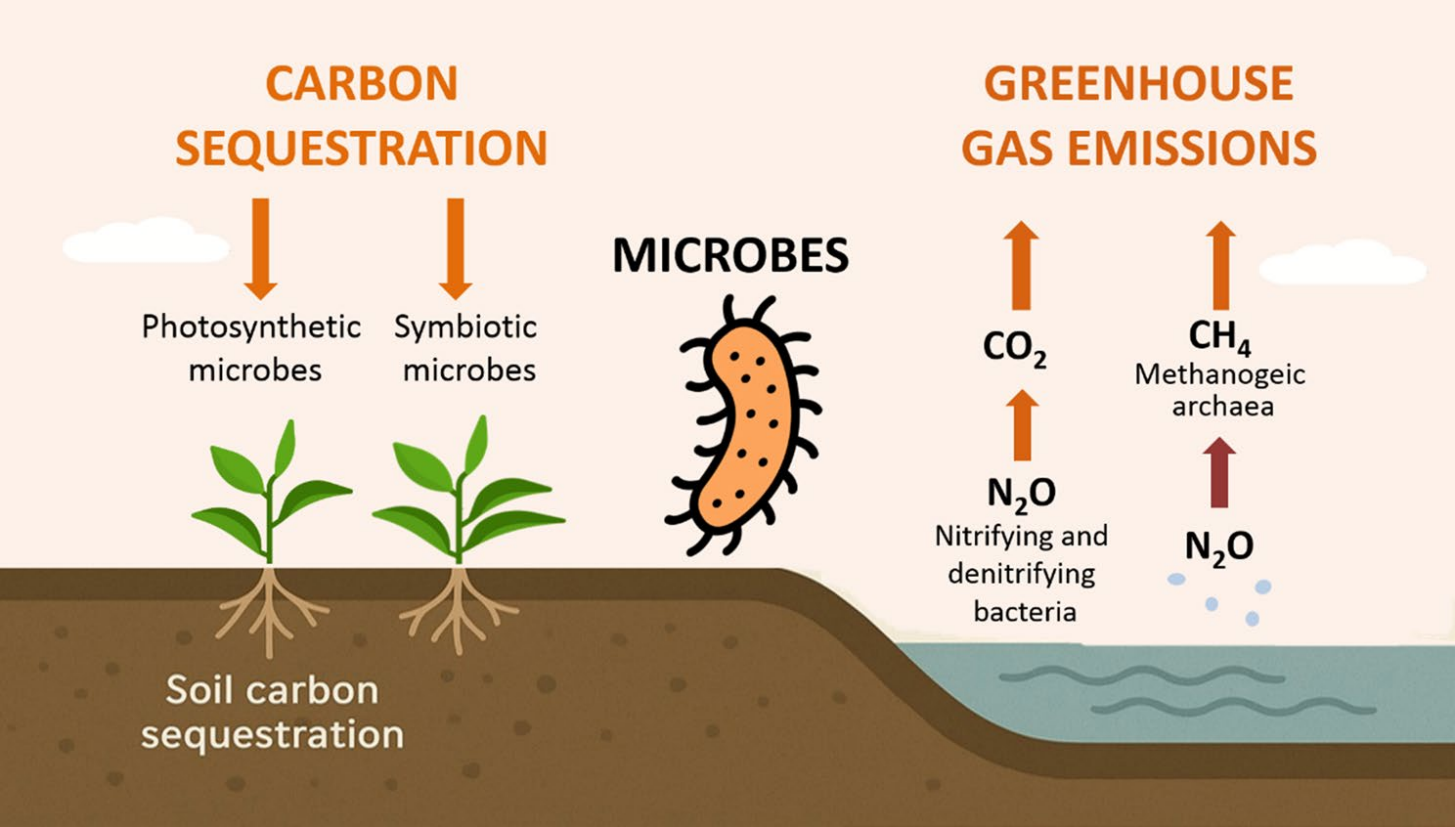
Microbial pathways of carbon sequestration and greenhouse gas cycling in soil ecosystems

The two primary global factors driving ecosystem change and biodiversity loss are urbanization and climate change [44]. Urbanization often reduces natural habitats to small, isolated patches surrounded by a built environment that is largely inhospitable to wildlife [45]. The continuous urbanization process negatively affects the urban environment, resulting in the urban heat island effect, a characteristic microclimatic phenomenon. Urban areas’ increased near-surface temperatures are indicative of this influence compared to nearby rural regions [46]. Crucially, the same urban expansion damages soil ecosystems where nitrifying microbes regulate nitrogen cycles and sequester carbon, a natural climate buffer now being destabilized by impervious surfaces and chemical runoff (Fig. 2). Urbanization also exacerbates climate-driven disease spread by creating ideal habitats for vectors.

## Vector-borne and zoonotic diseases

Vector-borne diseases are illnesses transmitted by infected arthropods such as mosquitoes, sandflies, and ticks. The survival, reproduction, and transmission of vectors, pathogens, and hosts depend on specific environmental conditions. Changes in these conditions can profoundly influence disease transmission dynamics [47, 48]. Climate directly affects the biological traits of both vectors and the pathogens they carry. Fluctuations in temperature can modify vector populations, transmission cycles, and interspecies interactions, ultimately influencing the emergence of zoonotic diseases [49]. Water availability also plays a critical role in vector oviposition behaviors, as increased precipitation creates more suitable breeding sites, enabling vectors to develop and mature more rapidly [50].

In terms of geography, vector-borne infections are usually limited to areas where their vectors are found. Nevertheless, with global warming, vector-borne infection transmission is likely to parallel the expansion of transmitting vectors into new regions [51]. Several diseases, including dengue fever, West Nile fever, chikungunya, malaria, leishmaniasis, Lyme disease, and tick-borne encephalitis, have recently emerged in Europe due to the spread of their respective vectors [52, 53].

It is important to recognize the emergence of new infectious diseases brought on by the geographic spread of insect vectors. For example, the mosquito *Aedes albopictus* is considered a highly adaptable vector, capable of transmitting more than 22 different arboviruses as well as several parasitic diseases [54]. The rise of vector-borne diseases in areas like North America and Scandinavia has been largely attributed to the northward migration of vectors like mosquitoes and ticks [55]. The three mosquito genera most commonly involved in transmitting diseases to humans and animals are Aedes, Culex, and Anopheles. Mosquito-borne diseases can spread either through vector species’ spread into new areas or through the adaptation of invading pathogens to local native mosquito species [55, 56].

## Malaria

Malaria is the most serious parasitic disease affecting humans. It is transmitted by female *Anopheles* mosquitoes and caused by five species of *Plasmodium* parasites, accounting for an estimated 620,000 deaths in 2017, the majority of which occurred in Africa. Over 80 countries remain endemic for malaria, placing nearly three billion people at risk [56, 57]. Environmental factors such as topography, precipitation, and temperature significantly influence malaria transmission. Tropical regions with high humidity, warmer temperatures, abundant rainfall, and lower elevations provide optimal conditions for vector survival and reproduction. These forecasts underscore the global public health impacts of climate change and highlight the urgent need for enhanced vector management strategies and measures to mitigate climate change in order to reduce the risks of infectious disease [50, 58].

## Dengue

The most serious disease spread by mosquitoes around the globe is dengue. The pathogen is prevalent in tropical and subtropical regions. Dengue viruses belong to the *Flavivirus* genus, with *Aedes aegypti* serving as the primary vector, followed to a lesser extent by *Aedes albopictus* [57]. Each year, dengue is responsible for approximately 10,000 fatalities and 100 million symptomatic cases annually, affecting populations in over 125 countries [58, 59]. By mid-century, rising temperature and rainfall are expected to expand dengue transmission into new regions across Africa and the Americas [60, 61].

## West Nile virus

**Fig. 3:**
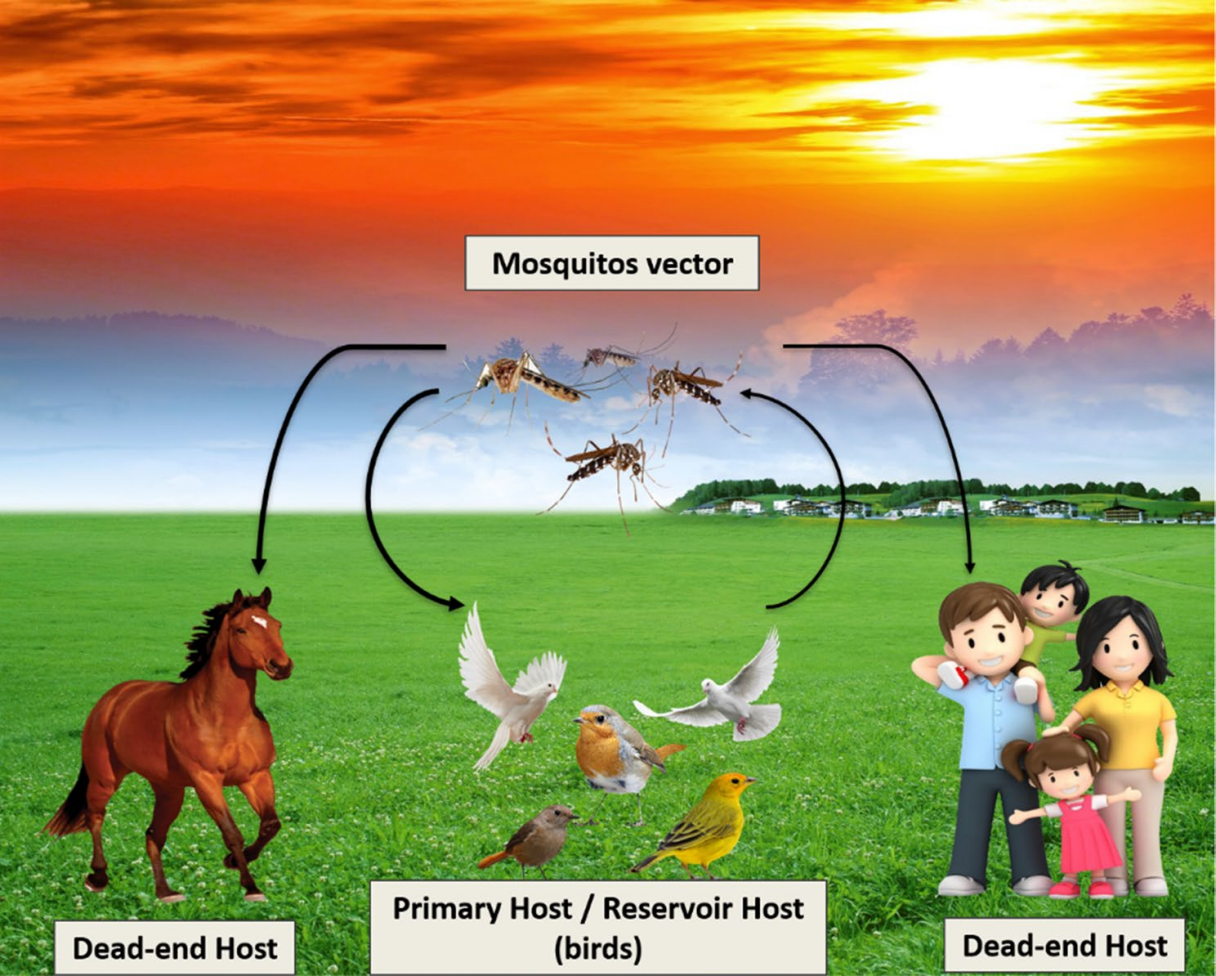
Transmission of West Nile virus

The *Culex* mosquito species is the primary vector of the West Nile virus (WNV), which can cause a spectrum of illnesses ranging from mild febrile conditions to severe meningitis or encephalitis [50]. According to [62], the primary hosts of these mosquitoes are wild migratory birds; However, transplacental or blood transfusion pathways have also been documented as transmission between humans, as illustrated in (Fig. 3).

Temperature is a key factor influencing WNV transmission, as optimal temperatures regulate the extrinsic incubation period, mosquito survival, and vector development rates [63]. These findings are particularly concerning because vectors can facilitate the spread of viral infection to new geographic areas. In 2018, the warmest year on record, Germany, which was previously thought to be non-endemic for WNV, reported the virus’s presence for the first time [64].

## Tick-borne disease

Ectoparasites such as ticks are distributed worldwide, and their eco-epidemiology is strongly influenced by environmental conditions. While feeding on their hosts, these hematophagous ectoparasites act as carriers or reservoirs for a wide range of pathogens, including harmful bacteria, viruses, protozoa, rickettsiae, and fungi. Despite the fact that ticks can spread a wider variety of diseases than other arthropod vectors, infections transmitted by ticks generally develop much more slowly compared to mosquito-borne infections [54, 55, 65].

## Lyme disease

In the Northern Hemisphere, Lyme disease is the most prevalent tick-borne illness, with an estimated 300,000 cases annually in the USA and 65,000 in Europe. The primary vectors of the spirochete bacterium *Borrelia burgdorferi*, which causes the disease, are the ticks *Ixodes pacificus* and *Ixodes scapularis* [66]. Like mosquitoes, ticks are greatly impacted by the weather, and climate change has been connected to the increased incidence and severity of Lyme disease. Warmer winters due to global warming have contributed to a significant increase in tick populations over the past decade [67]. High temperatures affect tick dispersal, life cycle progression, population density, and egg development. In Canada, studies predict a 213% increase in suitable tick habitats by the 2080s, with temperature identified as the most critical factor for the establishment of tick colonies [68]. In the USA, a 2 °C warming scenario is projected to extend the annual Lyme disease season, leading to a 20% rise in cases over the next few decades, with earlier onset and longer transmission seasons [58]. Aside from vector-borne diseases, climate change increases the prevalence of food- and water-borne infections, posing a significant global health burden. As rising temperatures and extreme weather disrupt microbial environments, both food and water safety are increasingly compromised.

## Climate-sensitive infections (food and water-borne diseases)

Food- and water-borne diseases pose a significant threat to global public health, with their incidence peaking during summer and increasing with rising temperatures and relative humidity [69]. Food-borne illnesses occur when contaminated or toxic food is consumed, and high temperatures can accelerate pathogen replication cycles and enhance their development, survival, and transmission. Climate change is projected to increase *Campylobacter* cases in countries like Norway, Sweden, Denmark, and Finland by the end of the century, with a 200% increase in cases in Norway, Sweden, Denmark, and Finland [50, 70]. Water-borne diseases, such as cholera, *Shigella*, *Salmonella*, *E. coli*, giardiasis, cryptosporidiosis, and viral hepatitis, are also triggered by climate fluctuations and changes. Cholera incidence rises with increasing water temperatures, while severe rainfall and flooding are linked to leptospirosis and other water-borne illnesses [71, 72]. Beyond acute infections, climate change also accelerates chronic and systemic threats such as AMR, further complicating disease control.

## Antimicrobial resistance and climate change

Microbes are essential to the cycling of nutrients and the maintenance of life. Their remarkable adaptability and functional versatility allow them to thrive in numerous environments, both synthetic and natural, and to adapt to fluctuating environmental conditions. A striking example of microbial evolution and adaptation is the growing crisis of the AMR epidemic, which poses a serious threat to public health [73]. Both AMR and climate change independently represent significant challenges to the health and well-being of plants, humans, animals, and ecosystems worldwide. Climate change, regarded as this century’s biggest threat to global health [74], is anticipated to result in more than 250,000 fatalities each year by 2050 due to heat-related illnesses, malaria, malnutrition, and diarrheal diseases [75].

## Climate-driven environmental stressors fuel AMR

Global warming and rising CO₂ levels pose severe threats to human health, future sustainability, and ecosystems. In the United States, local temperatures and population density are rising and have been correlated with increased AMR in common pathogens such as *E. coli*, *Staphylococcus aureus*, and *Klebsiella pneumoniae* [76]. Large-scale surveys, including those from Europe and China, confirm that higher ambient temperatures are associated with greater prevalence of antibiotic-resistant infections [77, 78]. Among these affected microbial ecosystems, marine and soil microbiomes are particularly vulnerable. Marine microbiomes, forming the foundation of ocean food webs and the global nutrient cycle, are predicted to undergo significant shifts due to rising ocean temperatures and increased acidification [79]. Similarly, rising global temperatures alter the structure of soil microbial communities, accelerating soil decomposition and the release of CO₂ [80, 81]. As bacteria adapt and become more tolerant to changing environments, in situ investigations of soil communities have shown that temperature and moisture have a significant impact on the function and composition of microbial communities, including the prevalence of pathogens and antimicrobial resistance genes (ARGs) [82–84]. Moreover, environmental stressors that drive declines in microbial diversity can also promote the environmental dissemination of ARGs [85]. Furthermore, extreme climate events such as floods can harm sanitation and healthcare infrastructure, promoting excessive antibiotic usage and spreading AMR. These risks are especially apparent in low- and middle-income countries, where vulnerable healthcare systems are already burdened by climate-sensitive illnesses and poor infection control capacity [86, 87].

## Environmental reservoirs of AMR

AMR persists and spreads mostly through the environment. Antibiotic residues from agriculture, healthcare, and industry provide strong selective pressures in soil, water, and wastewater systems [88]. Soil, one of the most diverse microbial habitats, harbor many resistance genes, including those relevant to clinical diseases [89–91]. AMR prevalence is considerably increased by practices like using animal or human waste and reclaimed wastewater to irrigate green spaces [92, 93].

**Fig. 4:**
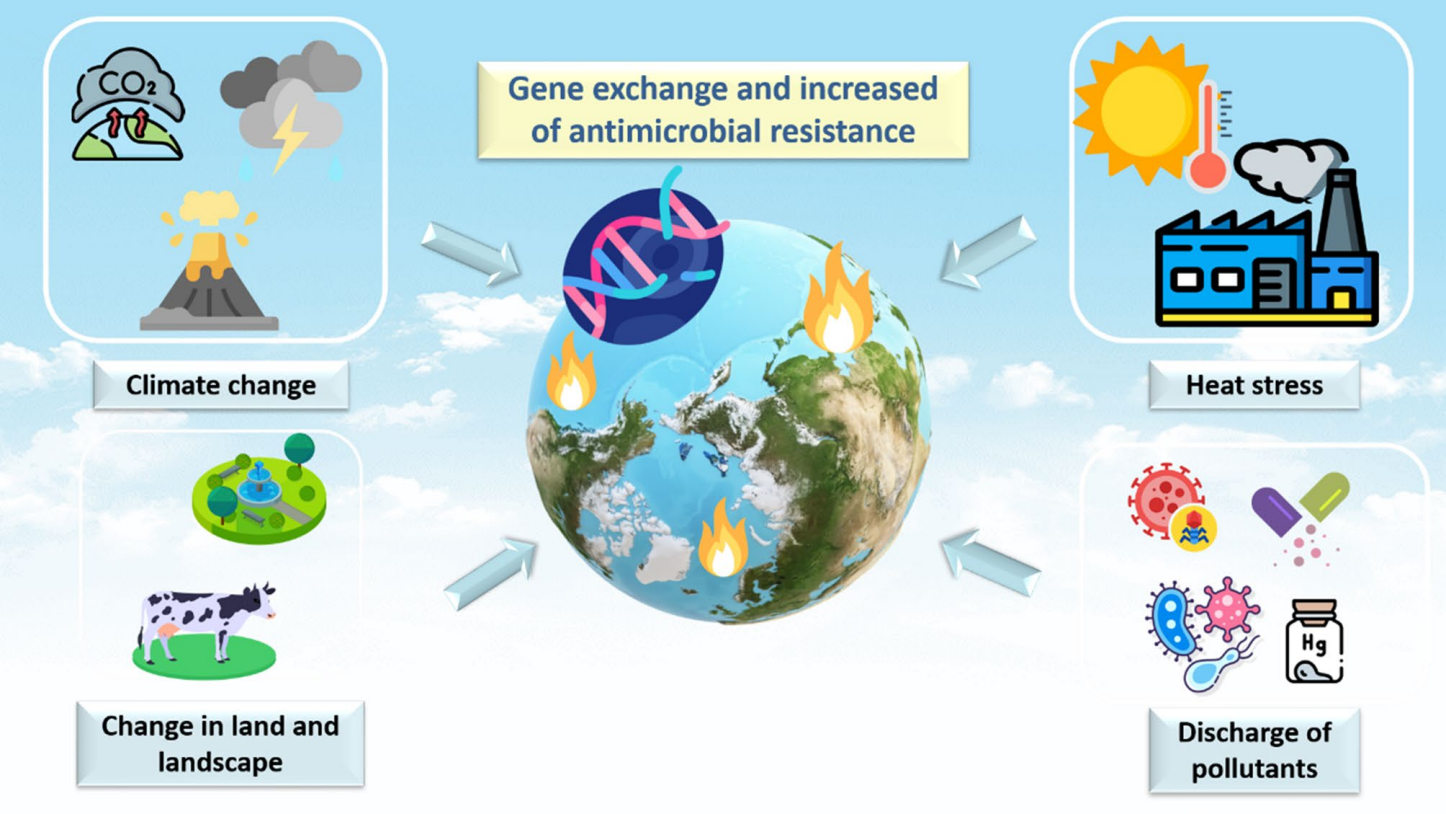
The effects of climate change on AMR development and transmission

Water and wastewater systems are similarly critical reservoirs. Although resistance genes naturally occur in aquatic habitats, their abundance has increased sharply due to anthropogenic contamination [86–88]. Even after treatment, resistant microorganisms and pharmaceutical residues persist in wastewater effluents, reinforcing their role as major sources of ARGs [94, 95]. Climate-related stressors, including rising water temperatures, salinity fluctuations, and pH shifts, further enhance microbial adaptation and horizontal gene transfer, accelerating the environmental spread of AMR [96–99]. At a broader scale, natural microbial communities are disrupted by the combined pressures of climate change, land-use alterations, and the release of pollutants such as heavy metals, antibiotics, and resistant organisms. These disruptions promote horizontal gene transfer and facilitate the circulation of resistance across ecosystems (Fig. 4) [73].

## Climate influence on antibiotic efficacy and microbial genetics

Beyond ecological dissemination, climate change directly affects microbial physiology and antibiotic effectiveness. Elevated temperatures alter gene expression and bacterial metabolism, reducing drug sensitivity. AMR prevalence has been positively correlated with warming: for example, carbapenem resistance in *Pseudomonas aeruginosa* increases 1.02-fold for every 0.5 °C rise [99]. Similarly, *E. coli* exhibits temperature-linked resistance responses across multiple antibiotic classes [100]. These findings highlight the complex interplay between environmental conditions, microbial evolution, and drug performance, reinforcing the need for One Health and systems-thinking approaches to mitigate climate-driven AMR risks [87]. Climate change not only affects environmental microbes, but it also alters the human microbiome, increasing vulnerability to metabolic, immune, and mental health disorders.

## Human microbiome disruption and modern diseases

The microbiome’s role in human health has grown significantly over the past decade due to advancements in techniques for studying complex microbial populations [101, 102]. Climate change threatens microbial habitats, ecosystem balance, and community composition, thereby raising the risk and severity of diseases. Understanding environmental conditions and climate change is crucial for preserving microbial diversity and its vital functions [103]. The microbiome is a crucial part of the human body as it plays an important role in multiple physiological processes, including digestion, immune function, metabolism, and mental well-being [2, 102, 104] The digestive system hosts a diverse community of microbes that influence nutrient distribution and assist in the production of essential vitamins and amino acids, including vitamin K and B [105, 106]. The interaction between humans and their microbiota is more than symbiotic, with gut bacteria fermenting non-digestible carbohydrates like dietary fiber, which generates short-chain fatty acids [107].

Climate change affects the gut microbiome, which includes beneficial bacteria like *Lactobacillus* and *Bifidobacteria*. Dietary shifts caused by climate change may lead to the emergence of harmful strains, posing a risk to public health [108, 109]. Although the gut microbiota can adapt to environmental changes and food shortages, dysbiosis has been linked to several health problems [103].

The respiratory microbiome, composed of various bacteria such as *Moraxella*, *Staphylococcus*, *Streptococcus*, and *Prevotella,* is also influenced by environmental factors that affect its composition and diversity, ultimately impacting respiratory health [110, 111].

The skin microbiome, which varies depending on the area of the body (oily, moist, or dry), is similarly affected by climate change. Dry areas typically host *Actinobacteria*, *Proteobacteria,* and *Bacteroidetes*, while oily areas are dominated by *Corynebacterium* [112–114]. Researchers have highlighted its defensive role against pathogen colonization through the production of antimicrobial peptides, which act as a protective barrier [115–117]. It also regulates sebum production, which helps in maintaing the skin hydration and resistance to adverse environmental factors [118]. The skin microbiome plays a vital role in maintaining immune balance and preventing autoimmune diseases by enhancing the responses of regulatory T cells [119, 120]. Infants require the development of beneficial microbes to build their immune system, preparing them fo\r environmental influences and climate changes [121]. A close interactive relationship between the gut and skin microbiome strongly reflects overall human health. Maintaining skin microbiome balance requires a diet rich in prebiotics and avoiding exposure to environmental pollutants.

**Fig. 5:**
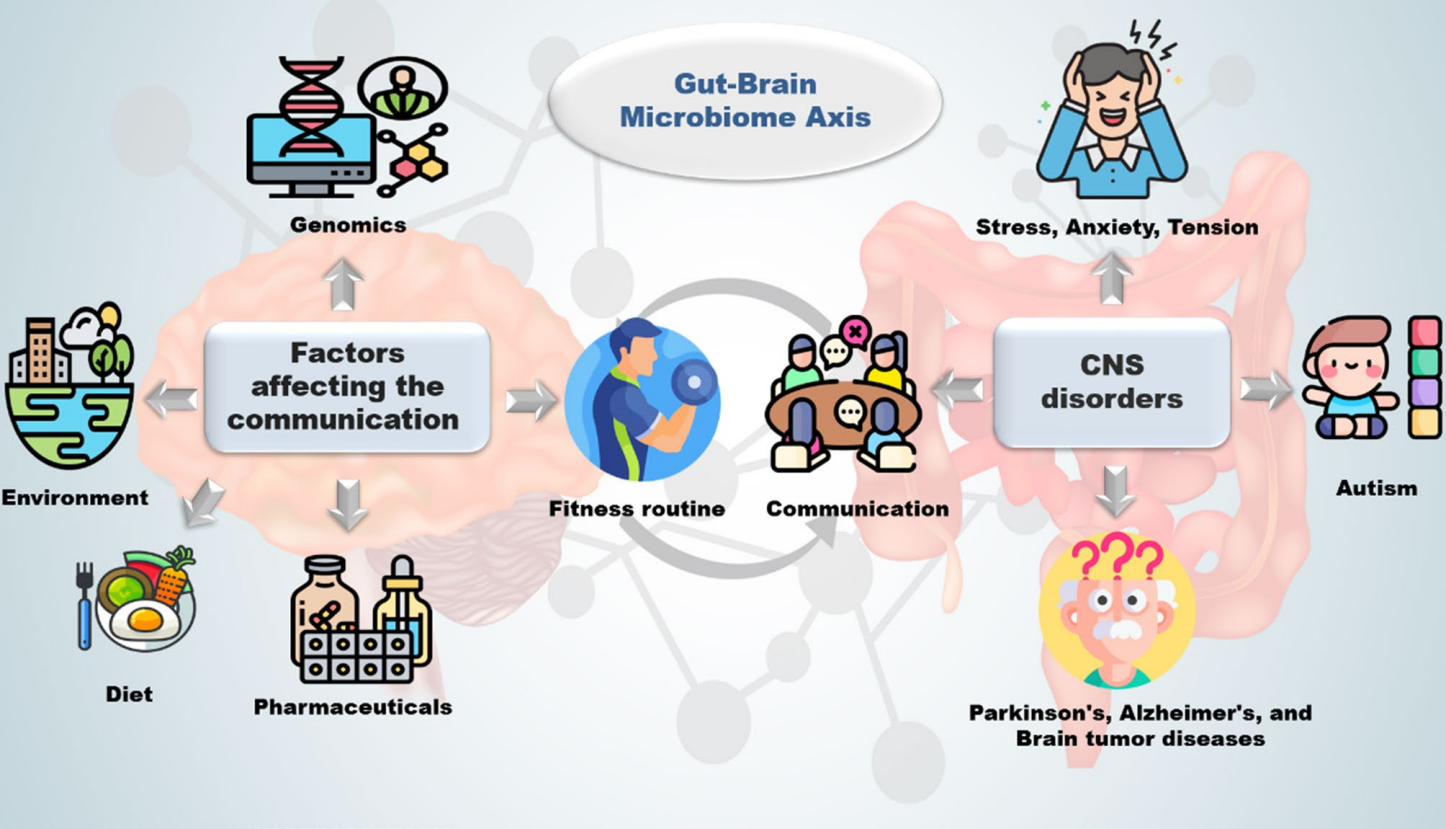
Gut brain–microbiome interplay and dysbiosis-linked disorders

The gut–brain axis, a bidirectional communication link between the gut microbiota and the central nervous system (CNS), is increasingly recognized as a key factor in mental health [122]. Through immune and neuroendocrine pathways, the gut microbiota regulates the CNS, influencing behavior, stress response, and emotional well-being. Conversely, CNS activity affects gut physiology and immunity [123]. Changes in gut microbiota have been associated with neurological disorders such as Parkinson’s and Alzheimer’s, as well as mental health conditions like anxiety and depression (Fig. 5) [124, 125]. Environmental factors, such as the excessive use of antibiotics, industrial and chemical waste production, urban expansion, deforestation, and fossil fuel use, contribute to microbial imbalance and low diversity, leading to a deteriorated immune system [126–128]. These factors cause complex immunological responses and change the human exposome [129]. Climate change, on the other hand, is predicted to increase the number of human pathogens by over 50% [130]. Increased intake of contaminated water during droughts may result in a greater enteric pathogen load and gut microbiota transformation, with water-borne pathogens like *Salmonella* becoming more prevalent due to extreme weather and heavy rainfall [131–133]. Air pollution, which is a significant environmental variable, induces oxidative stress in cells by generating reactive oxygen species, which overwhelm antioxidant defenses and damage DNA, proteins, and lipids [134, 135]. Genetic variants of glutathione-S-transferase enzymes, which protect cells from oxidative stress, have been linked to allergic diseases in populations exposed to air pollution [136].

The respiratory microbiome, which regulates host immune cells and forms a line of defense against pathogens, is negatively affected by any imbalance in the lung microbiome [137]. Studies have shown a link between lung cancer and respiratory diseases resulting from a microbial imbalance, with higher levels of *Streptococci* and *Prevotella* observed in lung cancer patients compared to healthy individuals [138–141]. Understanding the interactions between biodiversity and the skin microbiome is critical to controlling skin diseases, such as chronic skin infections. For instance, *Propionibacterium* is a common cause of acne [142, 143]. Dysbiosis, caused by an imbalance in gut microbes, can lead to diseases such as colorectal cancer, inflammatory bowel disease, and irritable bowel syndrome [144]. Obesity, diabetes, cardiovascular diseases, and autoimmune conditions, including asthma and eczema, are also linked to microbiome imbalances [145, 146]. Additionally, pesticides and chemicals used in crop cultivation are associated with inflammatory bowel disorders and colorectal cancer [147]. High temperatures can also negatively affect hepatic cells [148]. Just as antibiotic resistance spreads through disrupted ecosystems, so too does the collapse of microbial balance more broadly affect soil health, carbon cycling, and disease ecology. The collapse of microbial balance in response to climate-related stressors is inextricably linked to AMR. Both phenomena are driven by underlying factors such as gene transfer, microbial vulnerability to environmental shocks, and diminished ecosystem resilience. Thus, microbial imbalance should not be considered in isolation, but rather as a reinforcing cycle that accelerates resistance evolution and pathogen dissemination.

## Environmental shifts and the collapse of microbial balance

Climate change poses a significant threat to ecosystems and human societies, causing extreme weather events, natural disasters, and rising sea levels [149]. Microbial networks, influenced by climate change, adapt and reshape ecological dynamics [150]. Most ecosystems are at risk of extinction, and habitat change could cause catastrophic species extinctions depending on the species’ ability to adapt and migrate [151].

## Effect of temperature rise

Global warming affects CO₂ emissions from soil, negatively impacting microbial diversity due to nitrogen abundance [152]. This reduction in carbon use efficiency is critical for long-term soil carbon stability. Soil microbial communities have a crucial role in controlling climate-related soil activities by mediating important processes, including carbon sequestration and greenhouse gas cycling (Fig. 2). Temperature alteration also threatens aquatic organisms, leading to possible extinction [153]. Rising CO₂ levels in oceans reduce surface density and disrupt microbial food webs, with bacteria becoming more active and consuming large amounts of organic matter [154]. Ongoing climate changes will affect the balance of microbial and plant communities, selecting certain microbial species and potentially determining future ecosystem status [155, 156]

## Air pollution

Air pollution is the world’s largest environmental health concern. Exposure to air pollutants alters gut microbial composition and is associated with chronic diseases [157]. Anthropogenic factors such as pesticides, heavy metals, and wastewater also significantly impact microbial diversity and ecosystem function [158].

## Food security

Climate change severely threatens food security, reducing crop yields, quality, and nutritional value [159]. Microorganisms are essential for sustaining ecosystems, improving soil nutrient content, and suppressing harmful pathogens. However, climate change is disrupting these functions, contributing to food spoilage and disease development [160]. Rising temperatures and drought reduce agricultural productivity [161] and affect crop systems, forcing cultivation in unsuitable areas and necessitating drought-resistant microorganisms [162]. Climate change also increases pathogen spread, resulting in the emergence of new strains [27]. For example, drought can cause crop failures, pushing humans to consume processed, high-fat foods, which in turn alter the gut microbiome [163, 164]. Heat stress negatively affects meat and dairy cattle, decreasing yields and nutritional quality [165]. In poultry, heat stress induces oxidative stress and protein degradation, compromising product quality.

## Potential feedback loops between environmental microbiome shifts and human health

Climate change is causing self-reinforcing feedback loops that affect microbial biodiversity. Human-induced farming practices and the removal of trees and forests have impacted soil carbon concentrations, reducing plant microbial diversity and contributing to CO₂ emissions [166]. This deterioration impacts soil properties and biodiversity, emphasizing the need for soil preservation and studies to better understand microbial relationships [167].

Aquatic ecosystems are also affected by climate change, with rising water temperatures reducing phytoplankton presence and causing increased microbial growth, which contributes to higher CO₂ emissions [153]. Rising temperatures could disrupt biological processes and the geographic range of aquatic species, leading to the disappearance of cold-water species like salmon and trout while favoring the spread of warm-water fish species [168, 169]. Biodiversity protection and environmental ethics are crucial for sustainable coexistence. Understanding how these environmental disruptions destabilize microbial systems provides a foundation for exploring practical solutions aimed at restoring microbial balance and ecosystem resilience.

## Microbiome restoration strategies under environmental stress

Restoring microbial balance under environmental stress requires sustainable agricultural practices and targeted microbiome interventions, including probiotic and dietary strategies that support host resilience. The human microbiome plays a crucial role in health and disease, and recent studies indicate that probiotics can help balance the gut microbiome and prevent gastrointestinal infections.

Probiotics function by strengthening the epithelial barrier, producing antimicrobial compounds, competing for binding sites, and limiting pathogen access to nutrients. Diet plays a significant role in controlling microbiome activity and structure throughout life, with studies highlighting microbial colonization during infancy. Following a healthy diet supports balanced gut microbes in the face of environmental fluctuations. Addressing climate change and its environmental impacts is essential for future generations, who may face genetic changes impacting their disease susceptibility. These health threats are amplified in socioeconomically vulnerable regions, where climate-sensitive diseases intersect with weak infrastructure and inequity.

## Socioeconomic and healthcare system challenges in a changing climate

### The global south and the burden of a changing climate

Disparities in Climate-Vulnerable Regions (Global South Emphasis), extreme weather events, and rising temperatures significantly affect economic activities [170], reducing labor productivity, delaying investments, threatening human health [171], and disrupting ecosystem health and functionality [172]. Many individuals believe that the biggest threat to ecosystems worldwide is climate change [173]. The influence of climate change on marine biodiversity and ecosystem functioning has been extensively documented. These modifications result in changes to species physiology, population abundance, genetic structure, and interspecific interactions when paired with overfishing and coastal development [174]. Additionally, the African continent is projected to experience profound impacts on biodiversity, affecting both plant and animal species [173]. These conditions exacerbate social conflicts and negatively affect migration patterns, agricultural productivity, and community livelihoods [175]. As a result, ecosystems fail to provide the same essential services and benefits to society [176]. More specifically, the population of South Africa is particularly vulnerable to the consequences of climate change due to widespread poverty. It is expected that rising temperatures and decreasing rainfall would further lower crop production, placing approximately 25% of the population at heightened risk of diseases such as malaria, poor air quality, and food insecurity. Notably, air pollution accounted for 4% of total deaths in 2015 [177]. Savanna regions such as Ghana [178] and Kiribati [179] exhibit a similar trend of declining crop yields accompanied by rising malnutrition rates. During the 2015 drought, Ethiopia experienced a loss of 80% of its crops, leaving 8 million people food insecure, including 700,000 pregnant and lactating women and 40,000 children at substantial risk of malnutrition. This case illustrates how climate-driven shocks to food systems directly undermine nutritional security and heighten vulnerability to infectious diseases. Malnutrition compromises immune function, thereby amplifying susceptibility to microbial infections, while simultaneously straining already fragile healthcare infrastructures in climate-vulnerable regions [180].

Water quality in Pakistan is severely impacted by droughts, intense storms, and floods, all of which are associated with an increased incidence of infectious diseases such as cholera, typhoid, dengue, hepatitis, and malaria. Even in high-income countries like the UK, the effects of climate change are significant. The 2003 European heatwave caused a 25% decline in fruit harvests, leading to a spike in food prices. As healthy options became less affordable, consumers were pushed toward cheaper, ultra-processed foods. This illustrates how climate change can degrade nutritional quality and exacerbate noncommunicable diseases, such as obesity and cardiovascular disease, revealing a different, yet equally critical, facet of the climate-health feedback loop in industrialized settings [181]. In Canada, droughts are projected to impact water security, exacerbate respiratory conditions, and affect mental health. Moreover, they are likely to contribute to a rise in mental disorders, infectious diseases, and injuries [182]. In Italy, heatwaves have particularly harmed mental health, leading to an increase in suicides and psychiatric hospitalizations [183]. Amid these inequities, adaptive technological and policy frameworks are increasingly recognized as essential tools to mitigate climate-linked microbial and health risks.

## Building resilience through technology and policy

The growing incidence of climate-sensitive diseases underscores the close connection between global health and climate change [184]. For example, vector-borne diseases like dengue and malaria are spreading into previously unaffected areas due to changing precipitation patterns and rising temperatures, as previously mentioned. Similarly, respiratory diseases like asthma and chronic obstructive pulmonary disease are getting worse due to declining air quality and increased exposure to allergens like pollen [185]. Vulnerable groups, such as children, the elderly, and individuals with pre-existing medical conditions, are disproportionately affected, placing additional pressure on already strained healthcare systems [186]. These interconnected risks highlight the urgency of forward-looking, integrated solutions. In addition to direct health effects, climate change disrupts the foundations of healthcare delivery. Clinics and hospitals in disaster-prone areas face heightened risks of infrastructure damage, energy and water shortages, and supply chain breakdowns, all of which impede essential care [187–190]. To address these systemic vulnerabilities, a paradigm shift toward climate-resilient health systems is required, characterized by robust infrastructure, disaster readiness, sustainable resource management, and equity-centered design [191]. Technology offers powerful tools to complement system resilience. Artificial intelligence (AI), for example, is emerging as a transformative force in epidemic preparedness. By analyzing vast datasets, AI can identify anomalies, refine epidemiological models, and enable earlier detection of outbreaks [192–194]. When integrated with traditional surveillance and public health practice, AI strengthens the capacity to respond swiftly to zoonotic spillovers, AMR escalation, and climate-driven epidemics.

Finally, integrating climate and health policy frameworks ensures long-term sustainability. Embedding One Health and Planetary Health principles into governance can align climate mitigation, food and water security, and public health protection under a single agenda. In this way, AI innovation, resilient healthcare systems, and integrated climate–health policies form a solutions-oriented roadmap to mitigate microbial disruption and safeguard global health in a warming world.

## Outstanding research questions and future directions

There are still significant unknowns despite the rapid progress in our knowledge of the connections between microorganisms, health, and climate change. The ways in whereby climate-driven environmental changes upset microbial equilibrium and hasten antibiotic resistance should be better understood in future studies [195, 196]. Additionally, more research is required to determine the role that climate-induced microbiome dysbiosis plays in modern chronic diseases like diabetes, obesity, and mental health conditions. Lastly, the efficacy and equity of treatments, such as AI-based surveillance, sustainable agriculture, and probiotics, in vulnerable areas need to be thoroughly assessed. Answering these inquiries will be crucial for building climate-resilient, One Health–oriented strategies [197].

## Conclusion

As the planet heats, the effects of climate change go far beyond rising sea levels and harsh weather; they reach the invisible microbial world that supports all life. Climate change appears to be a strong driver of twenty-first-century health problems, changing pathogen evolution and accelerating the spread of antibiotic resistance, as well as upsetting the human microbiome and overwhelming fragile healthcare systems. These interlinked issues necessitate immediate, interdisciplinary action. Traditional compartmentalized approaches are no longer effective. A paradigm change toward integrated, One Health frameworks is required, acknowledging that the health of humans, animals, and ecosystems is inextricably intertwined. To anticipate and mitigate future biological threats, we must advance microbial research, build climate-resilient health systems, and use creative tools like AI. In the face of a rapidly changing globe, maintaining microbial balance across food, water, ecosystems, and the human body is more than just a scientific task; it is a global necessity. Looking forward, addressing these outstanding research questions will be critical for transforming awareness into action. By aligning scientific discovery with policy and equity, we can better safeguard microbial balance and human health in a warming world.

## Data Availability

No datasets were generated or analysed during the current study.

## Abbreviations

AMR: Antimicrobial resistance
WHO: World Health Organization
CO₂: Carbon dioxide
WNV: West Nile virus
ARGs: Antimicrobial resistance genes
CNS: Central nervous system
AI: Artificial intelligence
